# Extracellular Vesicles Derived from Bone Marrow Mesenchymal Stem Cells Protect against Experimental Colitis via Attenuating Colon Inflammation, Oxidative Stress and Apoptosis

**DOI:** 10.1371/journal.pone.0140551

**Published:** 2015-10-15

**Authors:** Jia Yang, Xing-Xing Liu, Heng Fan, Qing Tang, Zhe-Xing Shou, Dong-Mei Zuo, Zhou Zou, Meng Xu, Qian-Yun Chen, Ying Peng, Shuang-Jiao Deng, Yu-Jin Liu

**Affiliations:** Department of Integrated Traditional Chinese and Western Medicine, Union Hospital, Tongji Medical College, Huazhong University of Science and Technology, Wuhan, China; University of Torino, ITALY

## Abstract

The administration of bone mesenchymal stem cells (BMSCs) could reverse experimental colitis, and the predominant mechanism in tissue repair seems to be related to their paracrine activity. BMSCs derived extracellular vesicles (BMSC-EVs), including mcirovesicles and exosomes, containing diverse proteins, mRNAs and micro-RNAs, mediating various biological functions, might be a main paracrine mechanism for stem cell to injured cell communication. We aimed to investigate the potential alleviating effects of BMSC-EVs in 2,4,6-trinitrobenzene sulfonic acid (TNBS)-induced colitis model. Intravenous injection of BMSC-EVs attenuated the severity of colitis as evidenced by decrease of disease activity index (DAI) and histological colonic damage. In inflammation response, the BMSC-EVs treatment significantly reduced both the mRNA and protein levels of nuclear factor kappaBp65 (NF-κBp65), tumor necrosis factor-alpha (TNF-α), induciblenitric oxidesynthase (iNOS) and cyclooxygenase-2 (COX-2) in injured colon. Additionally, the BMSC-EVs injection resulted in a markedly decrease in interleukin-1β (IL-1β) and an increase in interleukin-10 (IL-10) expression. Therapeutic effect of BMSC-EVs associated with suppression of oxidative perturbations was manifested by a decrease in the activity of myeloperoxidase (MPO) and Malondialdehyde (MDA), as well as an increase in superoxide dismutase (SOD) and glutathione (GSH). BMSC-EVs also suppressed the apoptosis via reducing the cleavage of caspase-3, caspase-8 and caspase-9 in colitis rats. Data obtained indicated that the beneficial effects of BMSC-EVs were due to the down regulation of pro-inflammatory cytokines levels, inhibition of NF-κBp65 signal transduction pathways, modulation of anti-oxidant/ oxidant balance, and moderation of the occurrence of apoptosis.

## Introduction

Inflammatory bowel diseases (IBD), including ulcerative colitis (UC) and Crohn’s disease (CD), refers to a tract of chronic, idiopathic inflammatory disorders of the intestinal which are characterised by recurrent abdominal pain, protracted diarrhea, hardly cured stool with pus, blood and mucous[1]. Patients with IBD frequently experience relapse, and current medical therapies including corticosteroids, aminosalicylates and immunosuppressants, are not always able to keep patients in remission for a long term.

The first report of a clinical trial of cell therapy using autologous stem cells published in 2005 stated that stem cells were feasible and effective for the treatment of fistulas in Crohn's disease[2]. And then many other experiments got the similar conclusions that stem cell was effective in the treatment of IBD[3]. Our previous study also demonstrated that administration of bone marrow-derived mesenchymal stem cells could alleviate experimental colitis by modulating nuclear factor-κB-mediated pro-inflammatory response [4]. Thus may shed new light on the exploration of new approaches. However, as research continues, various studies have indicated these cells acted as potential sources of tumor associated fibroblasts (TAFs) [5] and have been found in many tumors including gastric lipoma[6], adenocarcinoma[7] and osteosarcoma[8], strongly suggesting their involvement in the process of tumor development. The application of BMSCs has been limited by the cancer risk.

Experiments based on stem cell transplantation revealed that only a small proportion of locally or systemically administered MSCs would actually be incorporated into injured tissues[9, 10], revealing that the beneficial effects in tissue regeneration and repair might probably depend on the paracrine activity of MSCs rather than their engraftment. The paracrine theory has recently changed the view of the biological action of stem cells and the subsequent potential application of stem cells in regenerative medicine[11]. Most of the paracrine physiological functions of stem cells have been attributed to the extracellular vesicles (EVs) they released [12]. EVs, released as exosomes from the endosomal compartment, or as shedding vesicles from the cell surface, have reported to have similar protective and ameliorative properties as the cells themselves in tissue repair [13–15]. Thus, MSC-EVs are likely to become a more efficient cell-free therapy approach that might overcome the obstacles associated with the use of native or engineered stem cells [16]. EVs has been demonstrated to protect the injure in various of tissue and diseases, including acute kidney injury[17], vascular injury[18], pulmonary hypertensionits [19] and Obesity [20], but its effect on colitis still remains vacancy. Consequently, we aim to investigate the potential alleviating effects of BMSC-EVs in colitis rats model induced by 2,4,6-trinitrobenzene sulfonic acid (TNBS) through anti-inflammatory, anti-oxidant and anti-apoptotic three aspects.

## Materials and Methods

### Ethics Statement

This study was performed in strict accordance with the Animal Research Institute Committee guidelines of Huazhong University of Science and Technology (HUST, Wuhan, China). The animal experiment was approved by the Institutional Animal Care and Use Committee (IACUC) of Huazhong University of Science and Technology. All of the animals experimental procedures were carried out in the experimental animal center of Huazhong University of Science and Technology (Permit number: SYXK(e)2010-0057). All efforts were made to minimize animal suffering.

### Animals

Male Sprague–Dawley (SD) rats weighed 160-180g were provided by the Center for Disease Control of Hubei province (Permit number: SCXK(e)2008-0005). Animals were housed under pathogen-free conditions at constant humidity and temperature (22–24°C), with 12/12 hr darkness-light cycles and free access to food and water.

### The cultivation and identification of BMSCs

Healthy SD rats weighted 80-100g were killed by cervical dislocation. The fur was disinfected with 75% alcohol and then the femurs and tibias were removed aseptically. Bone marrow cell suspension was obtained by flushing marrow cavity using dispensable 1-ml syringe with low-glucose dulbecco’s modified eagle medium (DMEM). Then bone marrow cell suspension was filtrated through 200-mesh sieve in order to remove bone debris. The obtained bone marrow cells were shifted into culture dishes and incubated at 37°C in a humidified atmosphere containing 5% CO2, with low-glucose DMEM plus 10% heat-inactivated fetal bovine serum (FBS, Gibco, Grand Island, NY, USA) and 100 000 U/L penicillin–streptomycin (Beyotime Biotechnology, Shanghai, China). The medium was changed every 3 days. Cells at 4^th^ to 6^th^ passage were utilized for subsequent experiments. Samples of cultured cells were authenticated on fluorescence-activated cell sorter (FACSAriaⅢ, Becton–Dickinson Biosciences, San Jose, CA, USA) to determine the expression antigen of mesenchymal stem cells with the use of anti-rat CD29, CD90, CD45 and CD11b (BioLegend, San Diego, CA, USA).

### The generation and identification of BMSC-EVs

The 4^th^ to 6^th^ passage BMSCs, 70% to 80% confluent, was used for the following EVs generation. BMSCs were thoroughly washed with PBS for three times and then incubated with serum-free low-glucose DMEM at 37°C for 48h. EVs were substantially generated from BMSCs when they were stimulated, such as starved by serum-free media to induce apoptosis [18]. EVs were obtained from supernatants following the method of Stefania Bruno et al [21]. Briefly, the cultured supernatants of BMSCs were centrifugated at 2000g for 20 mins in order to remove cells and debris. Cells free supernatants were centrifuged (Beckman Coulter Optima L-100K ultracentrifuge, Fullerton, CA, USA) again at 100 000g for 1 h at 4°C. The white precipitations in the bottom of the centrifuge tube were the EVs. The obtained EVs were washed in sterile PBS and submitted to a second ultracentrifugation in the same conditions. Then EVs were observed under 100kv transmission the electron microscope (HITACHIH-7000FA, Tokyo, Japan) to identify its morphology. Their size was analyzed by flow cytometry with the use of 1μm mean size red fluorescent beads (Sigma-Aldrich, St. Louis, MO, USA). The amount of EVs was tested by measuring the total EV-associated proteins using the Bradford protein assay (Beyotime Biotechnology, Shanghai, China).

### Experimental design and treatment protocol

After an adjustable feeding period of 1 week, male SD rats were randomly assigned to five groups: control group, TNBS group, 50μg EVs group, 100μg EVs group and 200μg EVs group (n = 10 for each group). Each rat was anaesthetized by intraperitoneal injection of chloral hydrate after a 24h fast. A soft polyethylene catheter of 2mm external diameter was inserted into the colon at the depth of 8 cm from the anus of rats. In TNBS group and the treatment groups, TNBS (Sigma-Aldrich, St. Louis, MO, USA) dissolved in 0.25 ml 50% solution of ethanol, was administered into the colon through the polyethylene catheter at a dose of 150 mg/kg body weight. In the control group, equal volume of PBS was used instead of TNBS to clyster. After instillation, the rats were held in a head-down position for 60s and then placed in the Trendelenburg position to prevent any liquid leakage. After awakening, the rats were given free access to chow and water again. On the third day after enema, the animals in 50μg EVs group, 100μg EVs group and 200μg EVs group received corresponding EVs dissolved in 1ml PBS via the tail vein injection respectively. The other two groups (the control group and the TNBS group) were injected with 1ml PBS instead. On the 7^th^ day after tail vein injection, all of the animals were anesthetized by intraperitoneal injection of 10% chloral hydrate and submitted to an aseptic laparotomy. We removed the entire colon and measured the colon length of each rats. The colon was opened longitudinally and slightly, and then rinsed in physiological saline to remove faecal residues. Tissue samples were store in liquid nitrogen for the subsequent experiments. To track whether these EVs reach the colon in vivo following transplantation, 6 other rats received lipophilic red fluorescence dye PKH26 (Sigma-Aldrich, St. Louis, MO, USA) labelled EVs after TNBS clyster. The PKH-26 staining process was accomplished according to the manufacturer’s manual before intravenous injection. The laser scanning confocal microscope (Olympus-FV1000, Tokyo, Japan) was used to observe the tissue slices of colon.

### Assessment of colitis

The score of DAI and macroscopic damage was evaluated by independent observers who were blind to the treatment. DAI was considered as a complex evaluation of body weight loss and the stool characteristics. The severity of the colitis was scored on a scale of 0–12 in accordance with the established criteria described by Hany H. Arab et al [22](Table 1). The macroscopic damage of colon was scored on a scale of 0–5 according to the criteria described by Galvez et al [23].The entire colon of each animal was rinsed with cold physiological saline to remove feces. The colon damage was examined visually and immediately. Briefly, macroscopic damage was scored as 0, for no damage; 1, for hyperemia and no ulcers; 2, for linear ulcer with no significant inflammation; 3, for Linear ulcer with inflammation at one site; 4, for two or more sites of ulceration or inflammation and the area extending<1cm; 5, for two or more major sites of ulceration or inflammation extending>1cm. Following macroscopic pathologic assessment, the fresh distal colon specimens were immediately fixed in 4% formalin and embedded in paraffin. Specimens were sectioned into 5μm and stained with hematoxylin and eosin (H&E) for histological analysis by light microscopy (Nikon TE200-U, Tokyo, Japan).

**Table 1:** Scoring of disease activity index (DAI).

Score	0	1	2	3	4
**Weight loss**	<1%	1–5%	5–10%	10–15%	>15%
**Stool consistency**	normal		soft		liquid
**Occult blood**	negative		positive		visible

### Biochemistry detection

The activities of SOD, MPO and the concentration of GSH, MDA in the colon were determined by the commercial biochemistry assay kit (Nanjing Jiancheng Bioengineering Institute, Nanjing, China) according to the manufacturer’s manual. SOD activity was assayed using the xanthine oxidase method based on the production of superoxide anion free radical. The absorbance was determined spectrophotometrically at 550 nm and the corresponding results of were expressed as U/mg protein. MPO was measured by colorimetric method based on the theory that one unit of MPO activity could degrade 1μmoL hydrogen peroxide at room temperature per minute. The results were expressed as U/g tissue; GSH concentration was determined on the reaction with 5, 5-dithiobis-(2-nitrobenzoic acid) (DTNB) and the absorbance was measured at 412 nm. GSH levels were expressed as μ mol/g tissue. MDA levels in the colonic samples were tested by the method of thiobarbituric acid (TBD). The absorbance was determined spectrophotometrically at 532 nm and MDA levels were expressed as n mol/g tissue. The experiments were repeated for twice.

### Elisa estimation

The levels of IL-10 and IL-1βin colon homogenate supernatants were measured using ELISA kits (Elabscience Biotechnology, Wuhan, China) employing the quantitative sandwich enzyme immunoassay technique. All the procedures were performed according to the manufacturer’s instructions. In brief, the samples were pipetted into the wells and incubated for 90 minutes at 37°C. Then the liquid of each well was removed and 100μL of Biotinylated Detection Ab working solution was added to each well immediately. After incubated at 37°C for 1 hour, each well was washed for three times and removed the liquid. Added 100μL of HRP Conjugate working solution to each well and incubated at 37°C for 30 minutes. Then repeated the washing process for five times. 90μL of Substrate Solution was added to each well and incubated in the dark for about 15 minutes at 37°C. The absorbance was read at 450 nm. The experiments were repeated for twice.

### Immunohistochemistry and immunofluorescent Staining

The fresh colon tissue samples from the rats were fixed in 4% paraformaldehyde, and then embedded in paraffin and cute into 5μm slices. The sections were rehydrated in xylene and dehydrated in grade concentrations of ethanol. Take these tissue sections in boiling citrate buffer (pH 6.0) for 10 min, and then treated with 3% hydrogen peroxide to inactivate endogenous peroxidase activity. After be blocked with 3% bovine serum albumin (BSA) for 30 min, the slides were incubated with primary antibodies overnight at 4°C for rat anti-NF-κBp65(Santa Cruz Biotechnology, Santa Cruz, CA, USA), anti-TNF-α(Santa Cruz Biotechnology), anti-iNOS antibody(Cell Signaling Technology, Danvers, MA, USA), anti-COX2 antibody(Cell Signaling Technology) and anti-caspase3 antibody(Bioworld Technology, Louis Park, MN, USA) respectively, followed by a secondary antibody, using DAB as the substrate. The nuclei were counterstained with Harris hematoxylin. For immunofluorescent staining, dewaxed sections were preincubated in PBS containing 3% BSA for 30 minutes. Sections were then counterstained with DAPI, washed and mounted with anti-fading medium. The location of PKH26 labeled EVs in the intestine were examined with microscopic examination using a laser confocal scanning microscope (Olympus-FV1000, Tokyo, Japan).

### Quantitative polymerase chain reaction (PCR)

Total RNA was extracted from colon samples using TRIZOL Reagent (Invitrogen, Carlsbad, CA, USA) according to the manufacturer’s protocol. Revert Aid First Strand cDNA Synthesis Kit (Thermo Fisher Scientific, Waltham, MA, USA) was used to produce cDNAs by reverse transcription. Target gene fragments were amplified by quantitative polymerase chain reaction (PCR) technology, performed with Fast Start Universal SYBR Green Master (Roche Diagnostics GmbH, Mannheim, Germany) according to the manufacturer’s instructions. In brief, initial denaturation of 1 cycle at 95°C for 10 min, followed by amplification of 40 cycles at 95°C for 15s, 60°C for 1 min. The target gene expression levels for each individual sample were normalized to β-actin, which is an internal control gene. The relative expression was calculated using 2^–ΔΔCT^ method. The results were expressed as an n-fold difference relative to the control group. Each sample was detected for three times. The quantitative real-time PCR primers are shown below:

Rat NF-κBp65, 5’- GCTCCTTTTCTCAAGCCGATGT-3’ (forward),

5’-CGTAGGTCCTTTTGCGTTTTTC-3’(reverse);

TNF-α,5’- CCAGGTTCTCTTCAAGGGACAA-3’ (forward),

5’- GGTATGAAATGGCAAATCGGCT-3’(reverse);

iNOS, 5’-CTTGGAGCGAGTTGTGGATTGT-3’ (forward),

5’- GGTAGTGATGTCCAGGAAGTAGGTG-3’ (reverse);

COX2, 5’-GCTTCGGGAGCACAACAGAG-3’ (forward),

5’- CAGCGGATGCCAGTGATAGAG -3’ (reverse);

β-actin,5’-TGCTATGTTGCCCTAGACTTCG-3’ (forward),

5’- TGCTATGTTGCCCTAGACTTCG -3’ (reverse);

### Western Blot

To further validate the expression of target protein observed from the immunohistochemical image, western blot was performed to quantify specific protein expression levels in the colonic tissues. Briefly, one volume of frozen intestine sample was homogenized in ten times the volume of RIPA lysis buffer (Goodbio Technology, Wuhan, China) containing a cocktail of protease inhibitors. After centrifuged the tissue homogenate at 12000g at 4°C for 10 min, the supernatants were collected and determined by Bradford protein assay to detect the protein concentrations. Protein extracts were loaded on each lane in sodium dodecyl sulphate–polyacrylamide gel electrophoresis (SDS-PAGE) gels. The polypeptides were separated by electrophoresis and further transferred into polyvinylidene fluoride (PVDF) membranes. Each membrane was blocked in 5% skim milk and subsequently incubated overnight at 4°C with anti-NF-κBp65 (1:500; Santa Cruz Biotechnology), anti-TNF-α (1:1000; Santa Cruz Biotechnology), anti-iNOS antibody (1:1000; Cell Signaling Technology), anti-COX2 antibody(1:1000; Cell Signaling Technology), anti-Caspase3 antibody (1:1000; Bioworld Technology), anti-Caspase8 antibody (1:1000; Santa Cruz Biotechnology), anti-Caspase9 antibody (1:1000; Cell Signaling Technology) and anti-β-actin (1:1000;Goodbio Technology) respectively. After being washed in TBST for 3 times, each membrane was incubated with a secondary peroxidase-conjugated antibody at room temperature for 30 min. The bands were visualized using enhanced chemi-luminescence (ECL) detection system (Amersham Pharmacia Biotech, Piscataway, NJ, USA). Protein bands were measuring by optic densities using alpha Ease FC and normalized to β-actin for statistical comparison.

### Statistical analysis

Normally distributed variables are presented as mean ± SD. Comparisons of categorical variables were carried out using one-way ANOVA followed by Bonferroni’s Multiple Comparison Test. P<0.05 was assumed statistically significant. Statistical analysis was performed by the use of IBM SPSS Statistics version 20(SPSS Inc., Chicago, IL, USA).

## Results

### Characterization of BMSCs and BMSC-EVs

**Fig 1:**
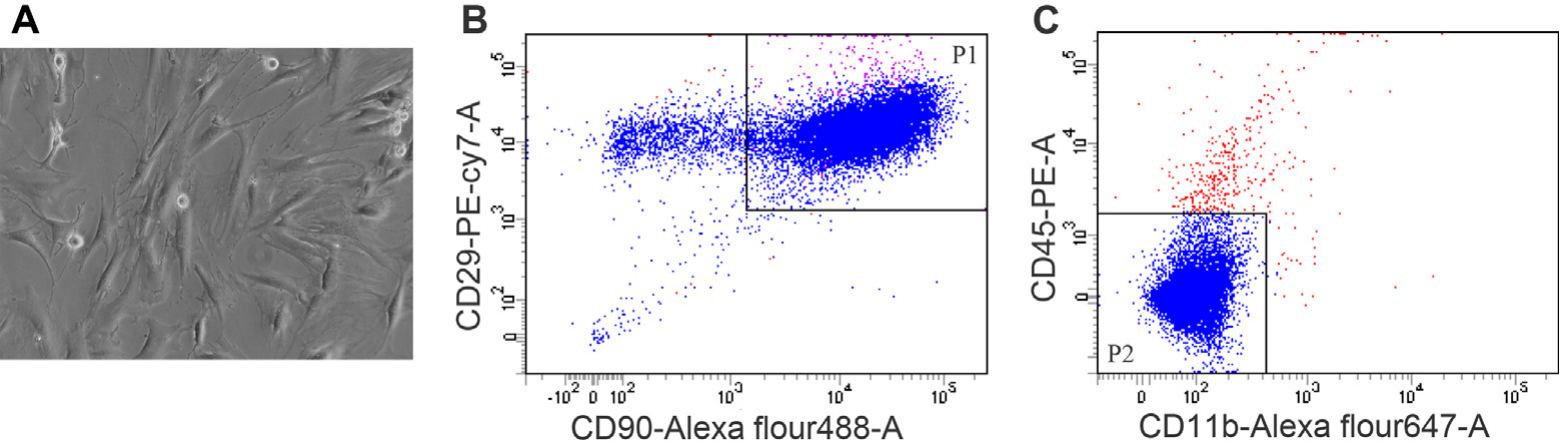
The morphology and identification of BMSCs. (A) Representative micrographs of photics microscopy obtained on BMSCs (original magnification ×200) at passage 4. (B and C) Characterization of BMSCs at passage 4 were evaluated by flow cytometric analysis using antibodies against CD90, CD29, CD11b and CD45. 89.8% (P1) of the BMSCs expressed surface markers phenotype of CD29 and CD90, while 97.5%(P2) did not have the lineage marker expression of CD11b or CD45.

**Fig 2:**
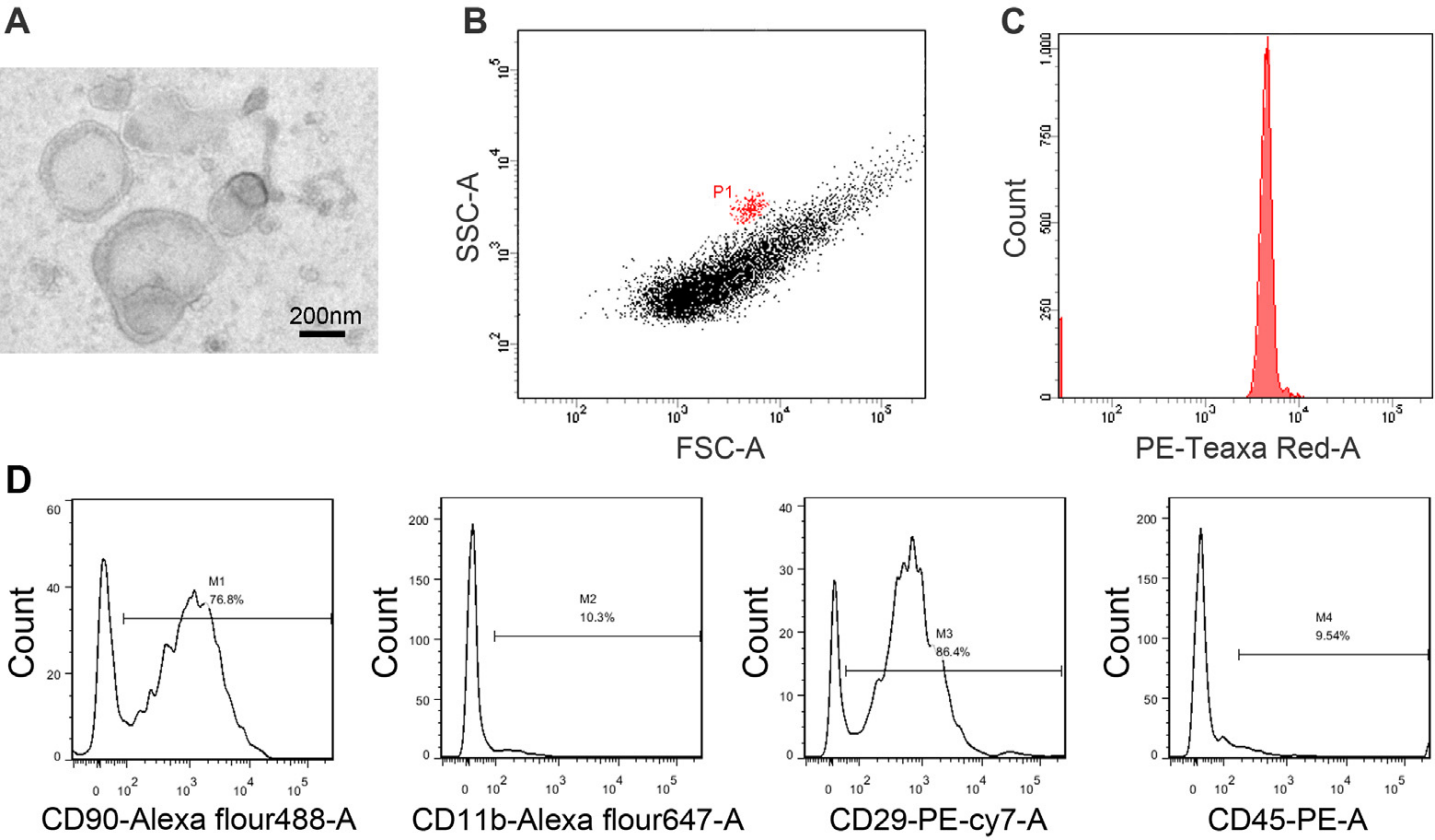
The characterization of BMSC-EVs. (A) Transmission electron microscopy analysis of purified EVs showed a spheroid shape. The black line refers to200 nm. (B and C) Flow cytometry was used to characterize the proper size of BMSC-EVs. Analyzed BMSC-EVs (black) were compared with Sulfate-modified polystyrene latex beads with a mean size of 1 μm (fluorescent red, λ_ex_ ~575 nm; λ_em_ ~610 nm). P1 represents the gate of the fluorescent beads. (D) FACS analysis of BMSC-EVs surface protein expression. The results indicated that BMSC-EVs were positive for CD90(76.8%), CD29(86.4%) and negative for CD11b(10.3%) and CD45(9.54%).

BMSCs were cultured as described in section ‘‘Materials and methods”. The cells were spindle-shaped and distributed as swirling under the microscope (Fig 1A). The makers of BMSCs were authenticated by flow cytometry (Fig 1B and 1C). There was a positive coexpression (89.8%, P1 part of Fig 1B) of bone marrow progenitor cell markers CD29 and CD90. While the double-negative expression for a panleukocyte marker CD45 and a monocyte/macrophage marker CD11b, accounted for 97.5% (P2 part of Fig 1C). The data demonstrated that BMSCs were cultured successfully. Purified EVs showed a spheroid shape under Transmission electron microscopy (Fig 2A). The bars indicate 200 nm. Flow cytometry was performed to further characterize the size of EVs (Fig 2B and 2C). Sulfate-modified polystyrene latex beads with a mean size of 1 μm (fluorescent red, λ_ex_ ~575 nm; λ_em_ ~610 nm) were used as reference beads. The images revealed that the diameter of majority EVs were less than 1μm. Flow cytometric analysis showed that BMSC-EVs (Fig 2D) exhibited positive staining for CD90(76.8%), CD29(86.4%) and negative staining for CD11b(10.3%) and CD45(9.54%), which were consistent with the BMSCs results. The Bradford results of EVs indicated that the total protein content was 1021.36±313.86μg/10^8^ BMSCs at the conditions of serum-free stimulation for 48h. The Bradford experiments were repeated for three times.

### Localization of BMSC-EVs after in vivo injection

**Fig 3:**
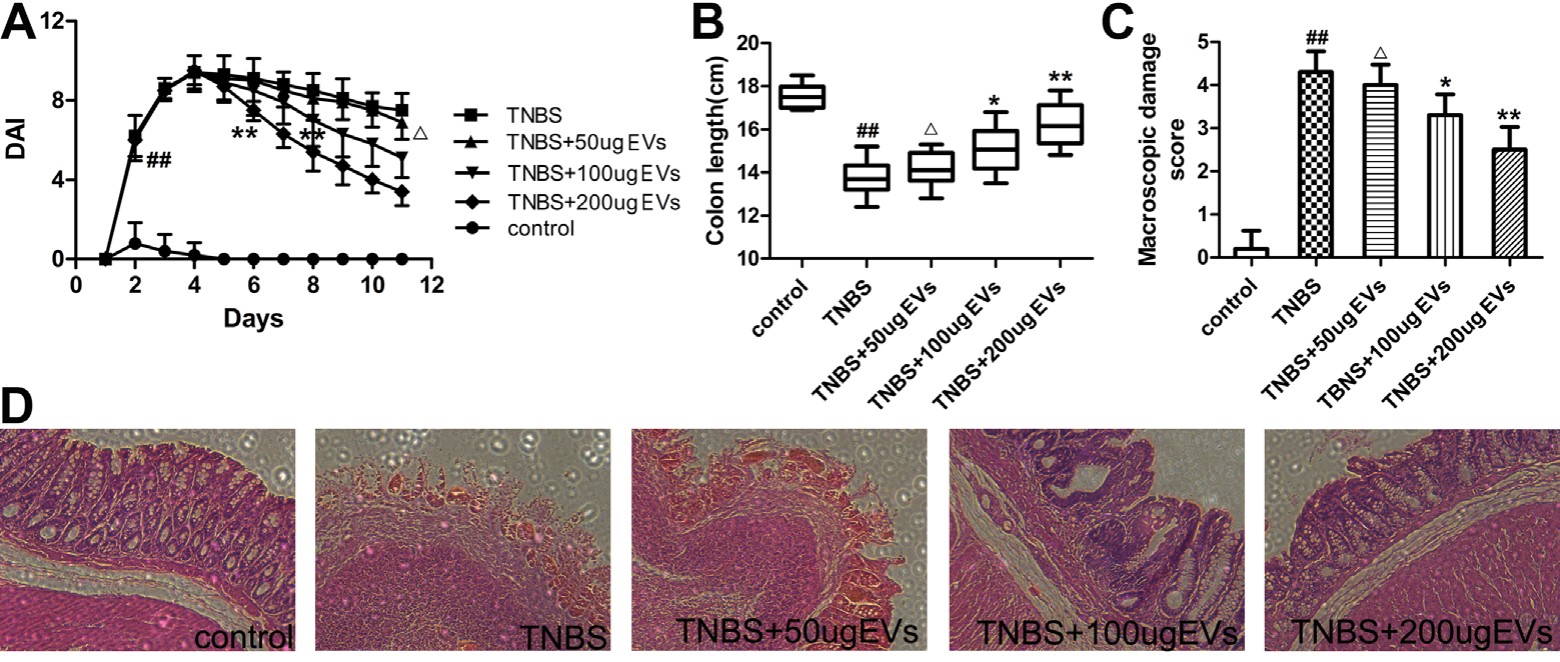
Confocal microscopy analysis of PKH26-labeled BMSC-EVs localization in colon after injection. The localization was measured 12h after intravenous injection. (A-I) Representative micrograph of inflammatory colon with the treatment of PKH26-labeled BMSC-EVs (red) at the dose of 50, 100 and 200μg. In merge images, nuclei were stained with DAPI (blue). Original magnification×200.

12 hours after PKH26 labeled BMSC-EVs were injected, rats were anesthetized and colons were removed under sterile technique. After being washed, freezing sections were made from these colon tissues. In the images, PKH26-labeled BMSC-EVs (red spots) were detectable in the cross-sections of TNBS induced colitis tissue shown by confocal microscopy. The results proved that EVs (Fig 3A–3I) could indeed reach the colon tissue which is a critical step in the protective effect.

### Effect of BMSC-EVs administration on the disease activity index and histological evaluation of rats with TNBS induced colitis

**Fig 4:**
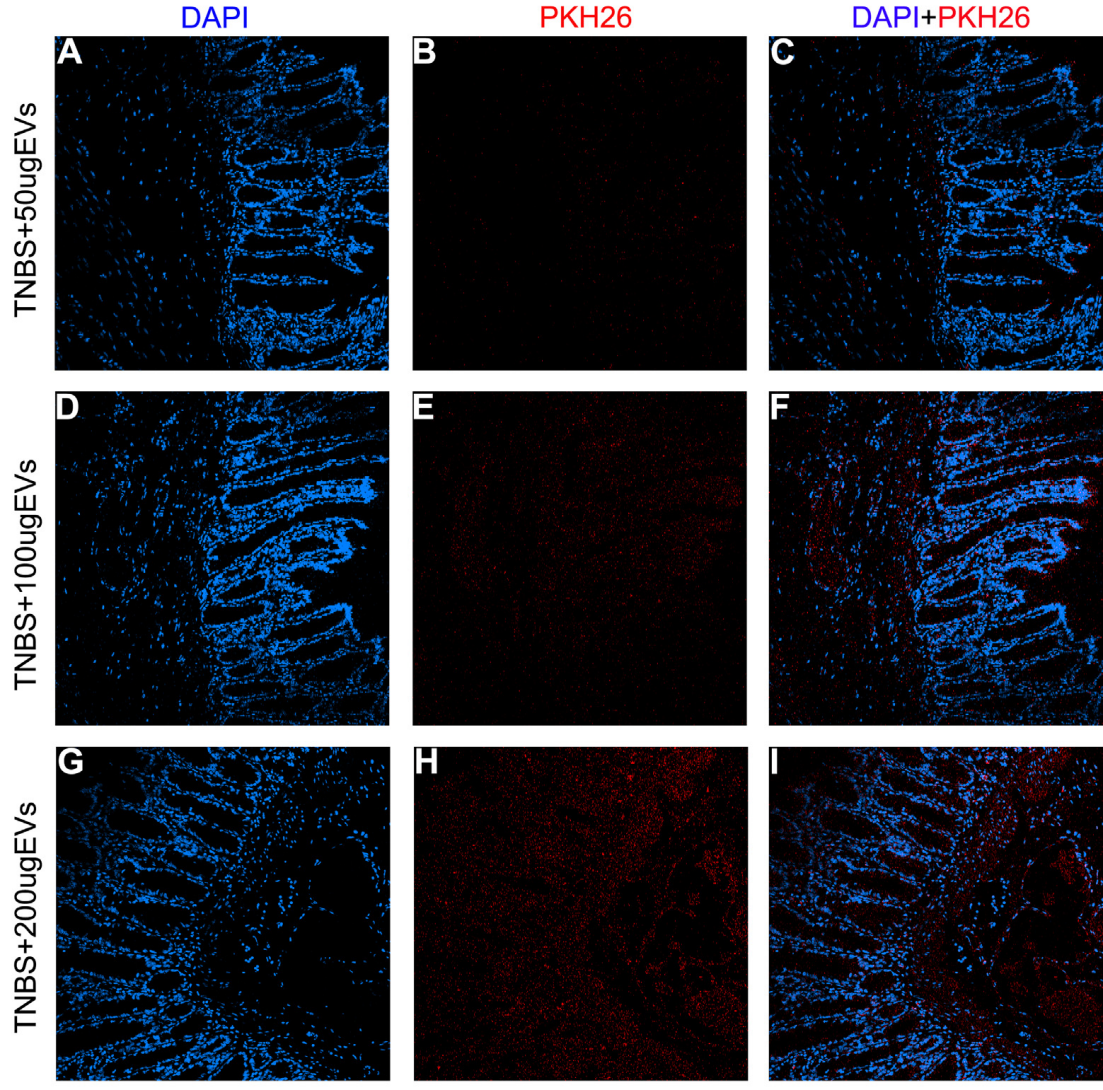
BMSC-EVs protect against TNBS-induced colitis of rats on symptoms and histological evaluation. (A) Change of clinical symptoms shown by disease activity index (DAI) of rats with TNBS-induced colitis. (B) Effects of BMSC-EVs administration on colonic length of rats with TNBS-induced colitis. (C) Effects of intravenously administered BMSC-EVs on macroscopic damage scores of colonic lesions with TNBS-induced colitis. ^##^P<0.01, vs control group. ^△^p>0.05, *P<0.05, **P<0.01 vs TNBS group. Values mean ± SD, n = 10 for each group. (D) Colon specimens were sectioned at 5μm and stained with hematoxylin and eosin (HE) for histological analysis (original magnification×400).

After TNBS enema, rats suffered a significant weight and appetite loss, slow activity, withered fur, severe diarrhea and even bloody stools. The DAI score (Fig 4A), utilized for estimate the severity of symptoms, was increased signally during the course of TNBS induction as compared with control rats (P<0.01). In contrast, BMSC-EVs treatment at the dose of 100 and 200μg were associated with gradual decrease of DAI score from day 4, compared with TNBS group. Decreased colon length is a reliable marker of colon inflammation. The intra-rectal instillation of TNBS observably reduced colon length (Fig 4B) as compared to the control groups (P<0.01). However, the administration of 100(P<0.05) and 200μg (P<0.01) BMSC-EVs significantly alleviate this change. Severe mucosal damage was observed in TNBS group, characterized by ulceration and inflammation. In the treated groups, with either 100 or 200μg BMSC-EVs, the damage was reduced, providing a significantly lower macroscopic damage score (Fig 4C) than that found in the TNBS group. The microscopic colon segments of rats showed the pathophysiologic structure by hematoxylin and eosin (HE) staining (Fig 4D). The character of inflammatory cell infiltration, massive bowel edema, transmural necrosis, focal ulceration of the colonic mucosa and epithelial cell disruption confirmed the successful establishment of colitis. BMSC-EVs treatment (at the dose of 100 and 200μg) indicated the amelioration effects on inflammation and ulceration.

### BMSC-EVs modulate inflammatory status in colon of rats with TNBS colitis

**Fig 5:**
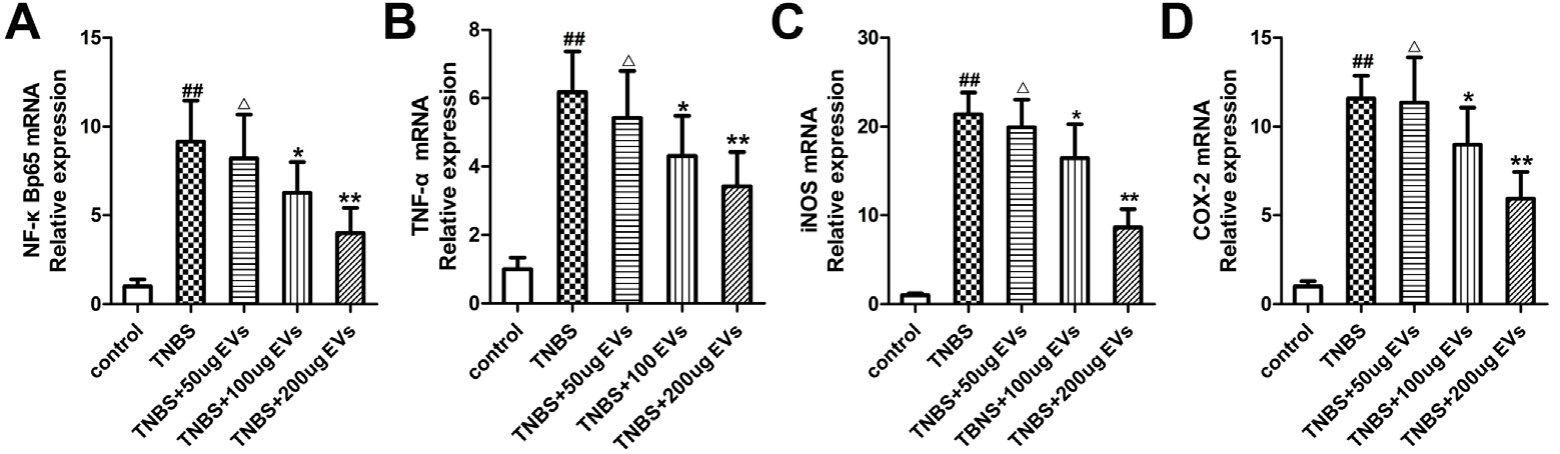
Effects of BMSC-EVs therapies on mRNA expression of inflammatory mediators in TNBS-induced colitis. The mRNA expression of NF-κBp65(A), TNF-α(B), iNOS(C) and COX-2(D) were detected by standard RT-PCR methods. β-actin was used as a control. Values mean ± SD, n = 10 for each group, differences are evaluated using the one-way ANOVA on ranks test. ^##^P<0.01, vs control group. ^△^p>0.05,*P<0.05, **P<0.01 vs TNBS group.

**Fig 6:**
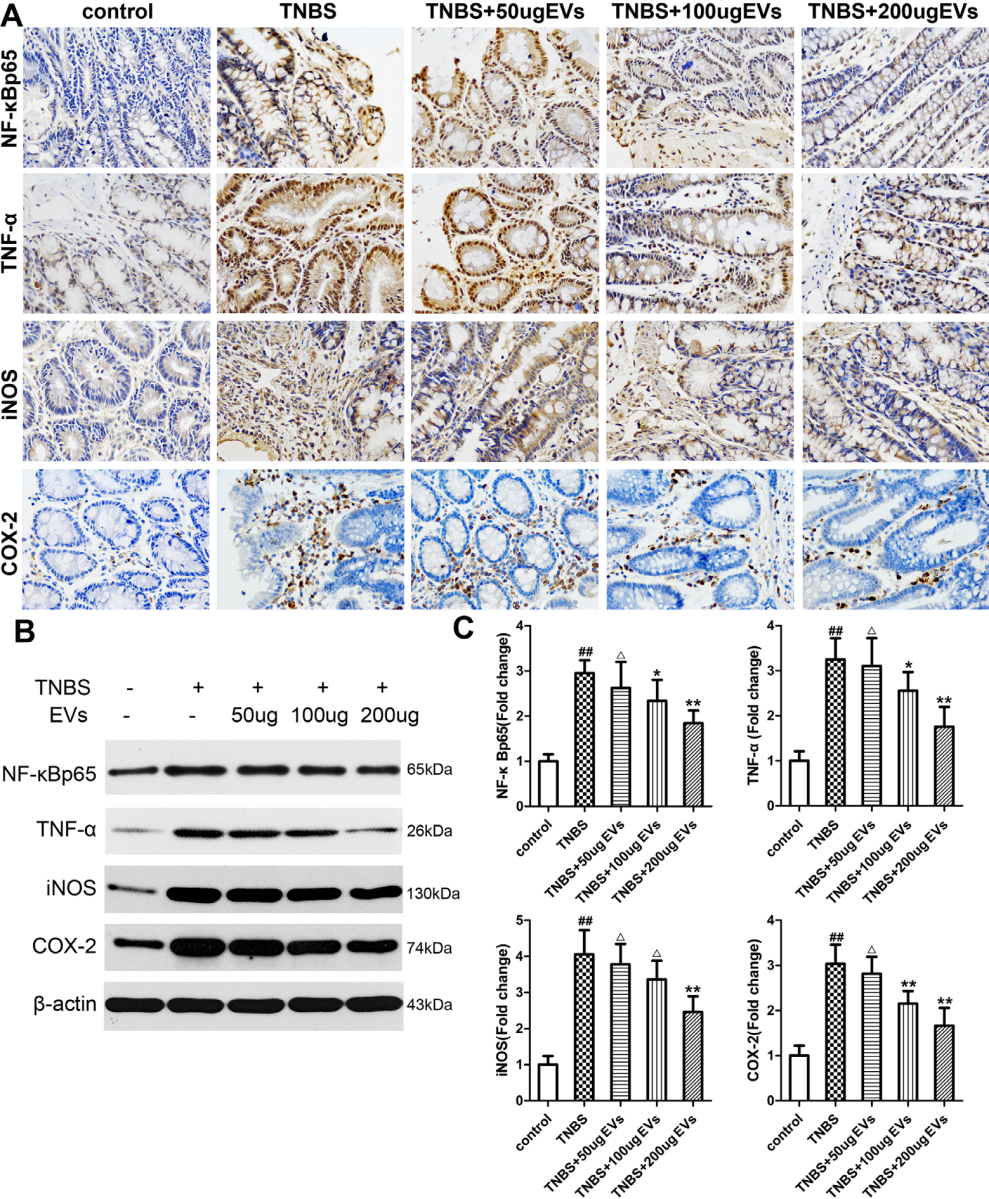
Effects of BMSC-EVs therapies on protein expression of inflammatory mediators in TNBS-induced colitis. (A) Immunohistochemical detection of NF-κBp65, TNF-α, iNOS and COX-2 protein expression. (B) Western blot detection of NF-κBp65, TNF-α, iNOS and COX-2 protein expression. (C) Grey value histogram of western blot detection. β-actin was used as a control. Values mean ± SD, n = 10 for each group, differences are evaluated using the one-way ANOVA on ranks test. ^##^P<0.01, vs control group. ^△^p>0.05,*P<0.05, **P<0.01 vs TNBS group.

**Fig 7:**
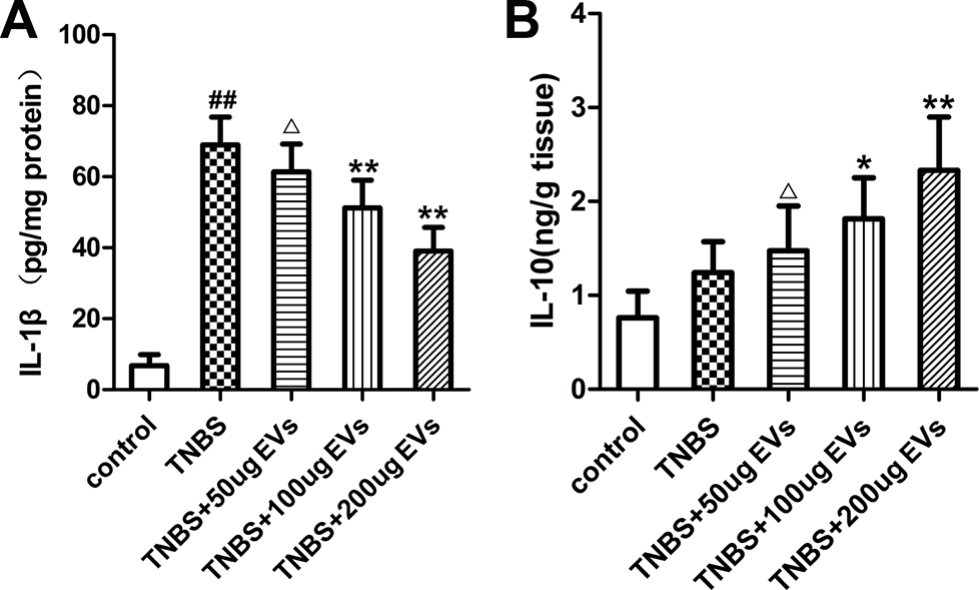
Effects of BMSC-EVs therapies on expression of IL-1βand IL-10 in TNBS-induced colitis. ELISA analysis was performed to detect IL-1β(A) and IL-10(B) expression. Values mean ± SD, n = 10 for each group, differences are evaluated using the one-way ANOVA on ranks test. ^##^P<0.01, vs control group. ^△^p>0.05, *P<0.05, **P<0.01 vs TNBS group.

To further investigate the mechanism of colitis on inflammation, we examined the expression levels of many inflammatory factors. As depicted in Fig 5A, TNBS administration increased NF-κBp65 subunit at the mRNA level (9.15-fold of the control levels) in colonic tissues whereas BMSC-EVs treatment (100 and 200μg) downregulated (P<0.05; P<0.01) its levels indicating a possible role of BMSC-EVs in attenuation of inflammation. We further extended our research to assess TNF-α (Fig 5B), iNOS (Fig 5C) and COX-2 (Fig 5D) mRNA expression which play crucial proinflammatory roles during the pathogenesis of IBD. In colitis group induced by TNBS, the data revealed significant increase (P<0.01) in the colonic expression of TNF-α(6.17-fold), iNOS (21.38-fold) and COX-2 (11.58-fold). In BMSC-EVs group, the mRNA expression of TNF-α, iNOS and COX-2 was reduced by 30.19%, 23.12% and 22.48% at the dose of 100μg, and 44.51%, 59.64% and 48.75% at the dose of 200μg, as compared to TNBS group (Fig 5B–5D). Accordingly, BMSC-EVs (100μg and 200μg) significantly decreased the protein expression of NF-kBp65, TNF-α, iNOS and COX-2, which were confirmed by immunohistochemistry (Fig 6A) and western blot analysis (Fig 6B and 6C). The similar tendency was observed in the protein level of IL-1β (Fig 7A) detected by Elisa. As to the anti-inflammatory substances IL-10(Fig 7B), Elisa analysis showed that colonic repair was characterized by a significant raise of 1.63-fold increase in TNBS group, 2.38-fold in 100μg BMSC-EVs group and 3.06-fold in 200μg BMSC-EVs group when compared with normal rats. These effects indicated the beneficial efficacy of BMSCs-EVs in downregulation the proinflammatory mediators and upregulation the anti-inflammatory mediators against colitis.

### BMSC-EVs inhibit oxidative stress and enhance colon antioxidant defenses

**Fig 8:**
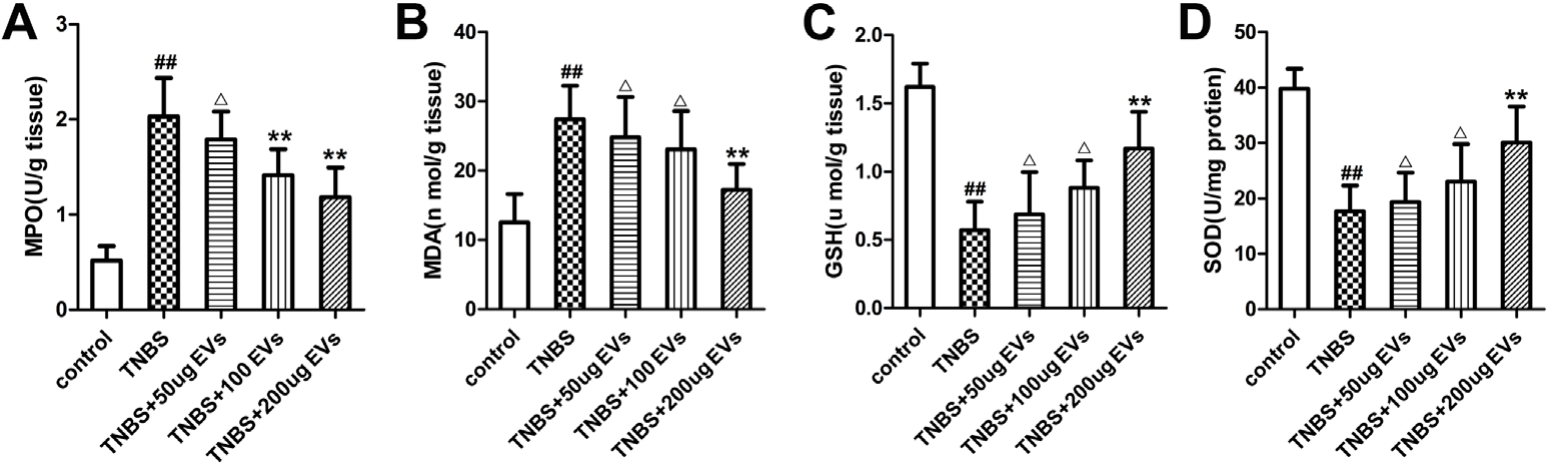
The effects of BMSC-EVs on oxidative stress and antioxidant defenses in TNBS-induced colitis. ELISA analysis was performed to detect MPO(A), MAD(B), GSH(C) and SOD(D) expression. Values mean ± SD, n = 10 for each group. ^##^P<0.01, vs control group. ^△^p>0.05,*P<0.05, **P<0.01 vs TNBS group.

To gain an insight into the therapeutical effect of BMSC-EVs on oxidative stress of TNBS colitis, we measured the levels of oxidants and antioxidants in colon tissues. As showed in Fig 8, enhanced oxidative stress was evidenced by remarkably increased levels of MPO(3.93-fold) and MDA(2.18-fold) along with decreased levels of GSH(64.75%) and SOD(55.56%) in colitis tissue, as compared to control group. Administration of 200μg BMSC-EVs displayed an effective inhibition in the activity of MPO(P<0.01) and MDA(P<0.01). Meanwhile, intravenous injection of 200μg BMSC-EVs also afforded significant antioxidant defense as verified by normalization of GSH levels(P<0.01) and SOD activities(P<0.01), as compared to TNBS colitis group. Together, these changes indicated the efficacy of BMSCs-EVs in attenuation of oxidative perturbations and boosting the colonic antioxidant defenses in the pathological process of IBD.

### BMSC-EVs down regulate the expression of apoptotic protein

**Fig 9:**
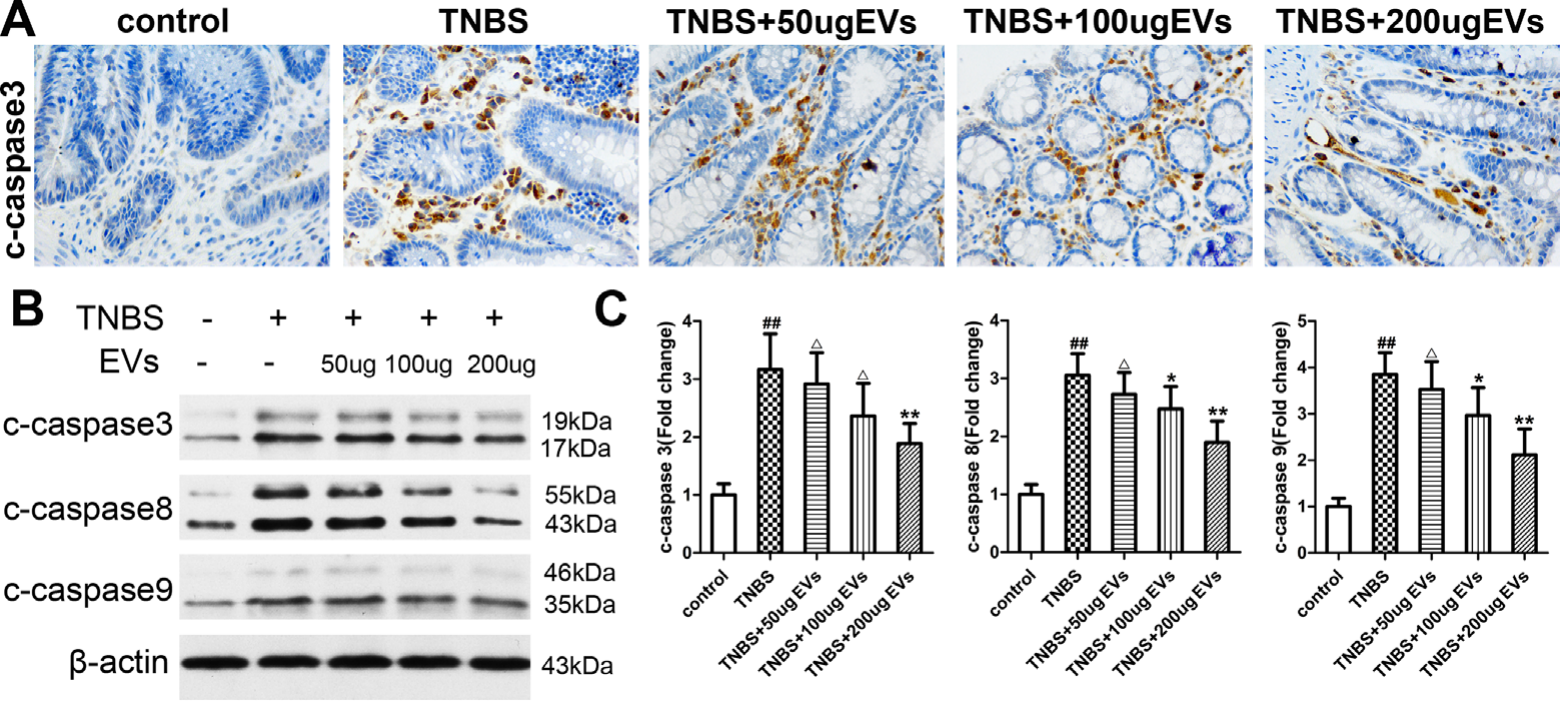
Therapeutic effects of BMSC-EVs on apoptosis in TNBS-induced colitis. (A) Representative micrographs of immunohistochemical localisation of c-caspase3 in colon mucosa at day 11(Original magnification×400). (B) Protein bands generated by Western blotting were performed to detect the protein expression of c-caspase3, c-caspase8 and c-caspase9. β-actin was used as a control. Protein extracts were obtained from colons. (C) Integrated optical density values of protein bands. Data are represented as mean ± SD of 10 animals of each group. ^##^P<0.01, vs control group. ^△^p>0.05,*P<0.05, **P<0.01 vs TNBS group.

We also assessed the activity of c-caspase3, c-caspase8 and c-caspase9, markers for colonic apoptosis, following the instillation of TNBS. Immunoreactivity for c-caspase3 was negative in colon sections of control rats. In comparison to the TNBS group, immunoreactivity was markedly reduced in rats with colitis receiving BMSC-EVs (Fig 9A). As expected, the result is consistent with Western blotting analysis of protein expression. The instillation of TNBS triggered apoptosis of inflamed colon as indicated by a 3.16-fold increase(P<0.01) of c-caspase3 protein expression(Fig 9B and 9C), a reliable indicator for apoptosis[24]. While tissues from inflamed colons displayed a 3.05-fold increase of c-caspase8 and a 3.85-fold increase of c-caspase9 activity as compared to control group, suggesting a marked activation of apoptosis in colon cells subjected to TNBS (Fig 9C). BMSC-EVs at the dose of 100 and 200μg inhibited the activity of c-caspase3 activity by 25.23% and 40.32% as compared to TNBS colitis group, and induced a significant down-regulation of caspase8 (18.97% and 37.85%) and caspase9(22.94% and 45.01%). Taken together, these results implied a role of BMSC-EVs for combating apoptosis and consequent loss of colon cells in colitis.

## Discussion

Bone marrow stem cell transplantation has been considered to have a potential therapeutic effect for tissue regeneration and organ repair in the treatment of certain degenerative gastrointestinal disorders including IBD[25]. However, the latent mechanism contributing to the tissue repair of allogeneic BMSCs in IBD has not been completely clear. In this study, we mainly focused on the therapeutic effect of BMSC-EVs, the major part of the paracrine mechanism of BMSCs, in experimental colitis induced by TNBS. We were surprised to investigate that EVs released from stem cells could provide a major contribution to the repair of colon, manifested in the mitigation of the bloody stools, weight loss, colon shortening, and microscopic injuries in acute colitis experiment by attenuating colon inflammation, suppressing oxidative perturbations and inhibiting apoptosis. Stem-cell derived EVs could be speculated to represent a relevant therapeutic option in regenerative medicine instead of stem-cell itself to a certain extent.

In the potential mechanisms for colitis, the NF-KB Signal transduction pathway and inflammatory mediators, including reactive oxygen species (ROS) and cytokines, such as interleukin-1β (IL-1β),inducible nitric oxide synthase (iNOS) and cyclooxygenase-2 (COX-2), produced by macrophages or epithelial cells, have been shown to contribute to the pathophysiology of colitis, especially in modulating the immune system of UC in the inflammatory cascade[26–28]. TNF-αelicits a wide spectrum of events such as differentiation, proliferation, inflammation and cell death [29]. In the amplification and prolongation process of inflammation, the over production of TNF-α, which is prevalent in the inflamed colon, has played an irreplaceable role [30, 31]. Local secretion of IL-1β, primarily coordinated by the activation of inflammasome, which is a protein complex that promotes inflammation and immunity[32], leads to local neutrophil recruitment[33] and Th17 cell differentiation[34] involved in IBD pathogenesis. COX-2 overexpression, essential to inflammation, injury repair, results in the production of ROS and excessive PGE2 and TXB2, which are important inflammatory mediators that contribute to the hyperemia, oedema and even dysfunction thereby inducing tissue damage. Similarly, iNOS activation produces an excessive inflammatory response, affecting to colon mucosa integrity and contributing to the development of intestinal damage[35, 36]. Our results showed that the expression of TNF-α, IL-1β, COX-2 and iNOS in colonic tissues was significantly higher in TNBS-treated rats compared to the control group, while BMSC-EVs treatment efficiently reduced the overexpression of these pro-inflammatory enzymes, playing a key role in the anti-inflammatory properties. Nuclear factor-κB (NF-κB), exists mainly as a heterodimer composed of p50 and p65 subunits, presented in an inactive state in the cytoplasm and activated via phosphorylation of IκBs by a variety of external stimulants [37]. It would be conceivable to infer that NF-kB could mediate the transcription of these proinflammatory genes, including TNF-α, iNOS and COX-2, since the promoter regions of these genes contain consensus binding motifs for NF-κB [38, 39]. In our study, Western blotting and RT-PCR results showed that the expression of NF-κBp65 observably up-regulated in TNBS induced colitis and decreased significantly in groups with the treatment BMSC-EVs (100 and 200μg). The results suggested that administration of BMSC-EVs effectively overwhelmed inflammation in the colon through the inhibition of NF-κB signal transduction pathways. IL-10 is one of the important anti-inflammatory cytokines, which could suppress T lymphocytes, mononuclear cell function and a large amount of pro-inflammatory cytokines [40]. Though the levels of IL-10 BMSC-EVs group significantly increased compared TNBS group, it is not forceful enough to contend with plenty of other inflammatory cytokines. Remarkable down-regulation of pro-inflammatory cytokines (such as TNF-α, IL-1β, COX-2 and iNOS) and up-regulation of anti-inflammatory cytokine(like IL-10) after using BMSC-EVs treatment (100 and 200μg) suggests that BMSC-EVs have an anti-inflammatory affect in TNBS-induced colitis linked with inhibition of NF-κB signaling pathways.

Under normal physiological conditions, a balance between oxidants and antioxidants keeps the normal function of the intestinal mucosa. Once the amount of oxidant production exceeds the capacity of endogenous antioxidant defense, associated with an increase in MAD and a decrease GSH and SOD, the gut would potentially be subject to injury [41, 42].Oxidative stress, as important as inflammatory affect, participated in the pathogenesis of inflammatory bowel disease, has been proposed to be related to the recruitment and activation of neutrophils infiltration of the inflamed colon mucosa during acute inflammation[32]. MPO is used to evaluate intestinal inflammatory processes as a biochemical marker of neutrophil infiltration [43]. It generates primarily hypochlorous acid and other reactive intermediates, leading to oxidative damage of lipids and proteins[44]. BMSC-EVs(100 and 200μg) led to reduced MPO activity in inflamed colon, pointing out an inhibition effect on granulocyte infiltration. Oxidative stress and its consequent lipid peroxidation are able to aggravate free radical chain reactions, disrupt the integrity of intestinal mucosa barrier, and activate inflammatory mediators, resulting in increased colonic MDA contents, as shown in experimental animal studies[45]. So the levels of MDA were often used as an indication of oxidative damage and a marker for free radicals-induced lipid peroxidation. The data in this study showed that BMSC-EVs(200μg) reduced MDA activity in TNBS-induced colitis, indicated that BMSC-EVs could act as the primary defense to reduce the oxidative stress. To counteract oxidative stress and keep cellular redox state in balance, intestinal mucosa possesses a complex of antioxidant systems. Superoxide dismutases (SOD), as a kind of enzymatic scavenger, could convert superoxide anion to hydrogen peroxide[46]. Studies also have shown that GSH protected the normal tissue cells against oxidative damage, reducing the sulfhydryl groups (-SH) of proteins and defending them from reacting with free radicals [47]. In our present study, the activity of SOD and GSH level in the TNBS group dwindled remarkably compared to the control group, while therapy with BMSC-EVs(200μg) resulted in a marked increase compared to the colon tissues of TNBS group. This result suggested that BMSC-EVs successfully inhibited oxidative stress in colitis induced by TNBS, reduced free radicals and strengthened enzymatic defensive system, then maintained the cellular oxidant/antioxidant balance.

The excess presence of TNF-α, oxidative stress and inflammation typically result in the occurrence of apoptotic effect in intestinal epithelial cells. We widely recognized that mammals cells subjected to apoptosis through two major pathways: the extrinsic death receptor (extrinsic) pathway and the intrinsic mitochondrial (intrinsic) pathway[48]. Caspase activity is a useful marker to detect apoptosis. The death receptor pathway is modulated by the recognition of extracellular ligands with transmembrane receptors such as Fas and TNF-related death receptors, which recruits the adaptor molecule FADD and caspase8 to the death-inducing signaling complex (DISC)[49]. In this regard, the occurrence of extrinsic apoptosis depends on activation of caspase8 and caspase3, without affecting the activation of caspase9. The intrinsic mitochondrial pathway is initiated by the cytochrome C release from the mitochondria. A variety of intracellular and extra stresses, including DNA damage, oxidative stress, heat shock and cytotoxic drugs, give rise to the release of cytochrome C from the mitochondrial intermembrane to the cytosol. Cytochrome C cooperated with apoptosis protease activating factor-19(Apaf-1) can promote the activation of caspase9 which could subsequently cleave and activate downstream effector caspase3 [50, 51]. The cleavage of a series of proteins, for example nuclear fodrin and lamins, caused by the activation of caspase3, could lead to cell apoptosis [52]. The TNBS enema led to a statistically clear increase in caspase3 activation which was revealed by the appearance of caspase3 cleavage fragment at 17 or 19 kDa. Similarly, the cleavages of caspase8 and caspase9 were also significantly enhanced by TNBS which further indicated that caspase-dependent apoptosis pathway may involve in the colitis pathological process. The result after tail vein injection of BMSC-EVs(100 and 200μg) showed that BMSC-EVs decreased the expressions of c-caspase9, c-caspase8 and c-caspase3. Our studies explored that BMSC-EVs could diminish cell death after TNBS enema in colon by inhibiting the intrinsic pathway manifested as the decrease in c-caspase9, as well as the extrinsic pathway evidenced by decrease of c-caspase 8. Taken the analyses together, our findings demonstrated that BMSC-EVs treatment was an effective anti-apoptotic agent in TNBS-induced colitis model through both the extrinsic death receptor signal pathway and the intrinsic mitochondrial signal pathway.

Transplanted BMSCs promoted tissue repair mainly by the paracrine mechanism [11, 16]. Some studies showed that the horizontal transfer of mRNA or microRNAs shuttled by EVs to the target cells was the mechanism of action [15, 16, 18, 20]. Recent studies[53] pointed out that RNA extracted from BMSC-EVs and cells of origin was profiled for 365 known human mature miRNAs and 41 co-expressed miRNAs in both EVs and cells. However, it remains largely unknown that which kind of specific mRNA/miRNA/protein released by BMSCs contributed to the biological activities of EVs. Therefore, to verify the relationship between mRNA/miRNA/protein shuttled by EVs and the protective effect of EVs will become the hotspot of EVs research in our future study.

## Conclusion

**Fig 10:**
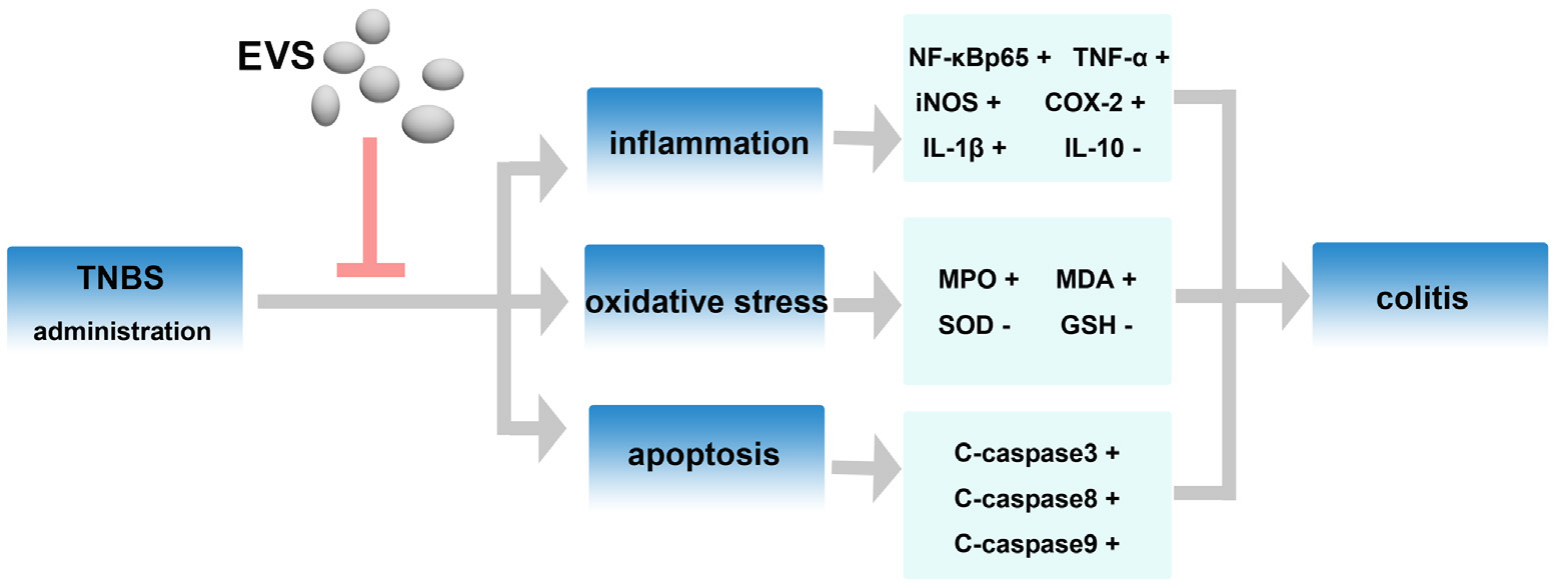
Diagram depicting the alleviating actions of BMSC-EVs in TNBS-induced colitis.

In conclusion, the current study provides evidences for the promising protective effects of BMSC-EVs in TNBS-induced colitis, manifested as modulation of inflammatory, suppression of oxidative stress and alleviation of apoptosis (Fig 10). EVs released from stem cells may confer a stem-cell-like phenotype and retain several biological activities to injured cells, reproducing the beneficial effects of stem cells and initiating the consequent activation of self-regenerative programmes in experimental model of colitis. Thus, our information provides new insight into the therapeutic effects of BMSC-EVs in colitis. Further studies are wanted to investigate the potential exact molecular (mRNA/miRNA/protein) shuttled by EVs that may influence the inflammation, oxidative stress or apoptosis.

## Supporting Information

S1 Data The data of DAI, colon length and macroscopic damage score. (XLSX)

S2 Data The data of ELISA. (ZIP)

S3 Data The data of biochemistry detection. (ZIP)

S4 Data The amplification curve of PCR. (ZIP)
