# Supplementary figures and images for: Extracellular Vesicles Derived from Bone Marrow Mesenchymal Stem Cells Protect against Experimental Colitis via Attenuating Colon Inflammation, Oxidative Stress and Apoptosis
Source: PLoS One. 2015 Oct 15;10(10):e0140551. doi: 10.1371/journal.pone.0140551 (PMC4607447; doi:10.1371/journal.pone.0140551)

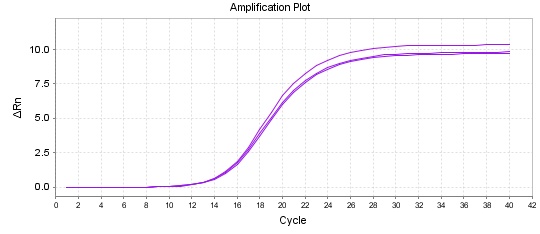

Supplement: S4 Data — (ZIP) [file pone.0140551.s004.zip › S4/ACTIN(100).jpg]

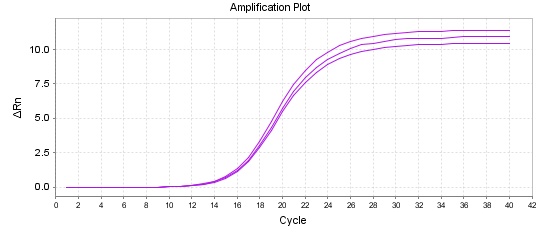

Supplement: S4 Data — (ZIP) [file pone.0140551.s004.zip › S4/ACTIN(200).jpg]

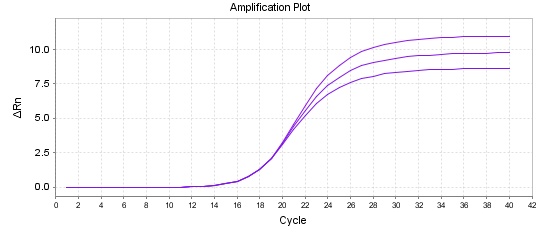

Supplement: S4 Data — (ZIP) [file pone.0140551.s004.zip › S4/ACTIN(50).jpg]

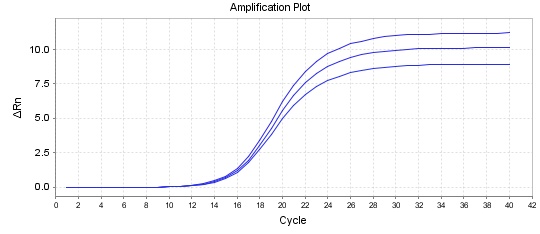

Supplement: S4 Data — (ZIP) [file pone.0140551.s004.zip › S4/ACTIN(control).jpg]

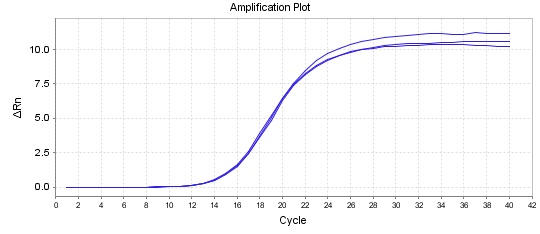

Supplement: S4 Data — (ZIP) [file pone.0140551.s004.zip › S4/ACTIN(tnbs).jpg]

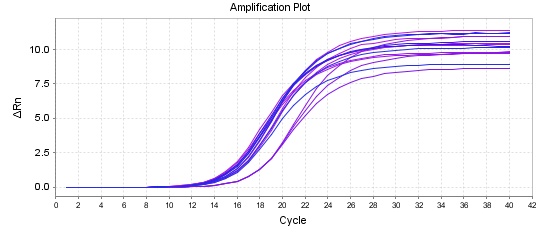

Supplement: S4 Data — (ZIP) [file pone.0140551.s004.zip › S4/ACTIN.jpg]

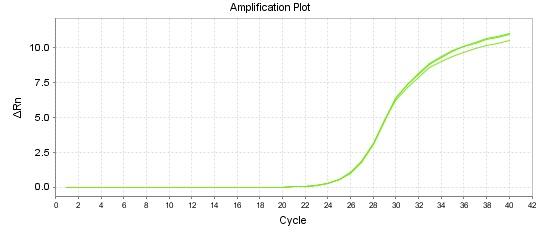

Supplement: S4 Data — (ZIP) [file pone.0140551.s004.zip › S4/COX2(100).jpg]

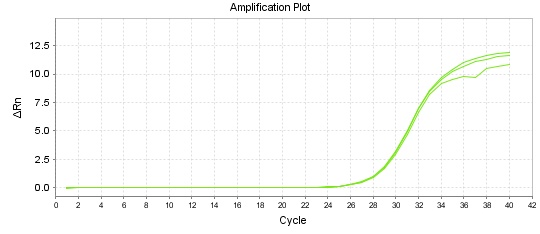

Supplement: S4 Data — (ZIP) [file pone.0140551.s004.zip › S4/COX2(control).jpg]

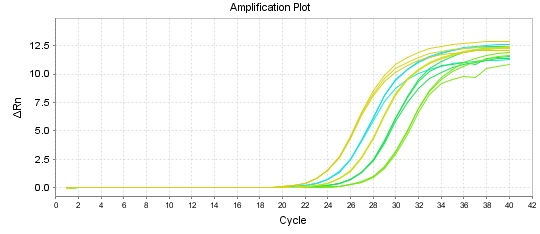

Supplement: S4 Data — (ZIP) [file pone.0140551.s004.zip › S4/COX2.jpg]

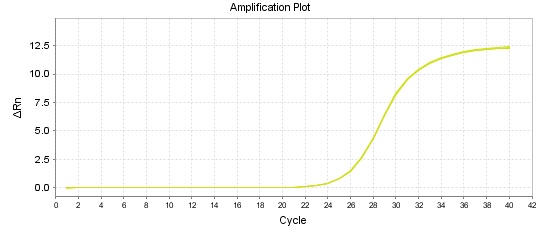

Supplement: S4 Data — (ZIP) [file pone.0140551.s004.zip › S4/COX2ú¿200).jpg]

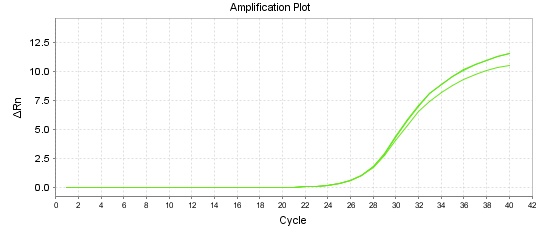

Supplement: S4 Data — (ZIP) [file pone.0140551.s004.zip › S4/COX2ú¿50).jpg]

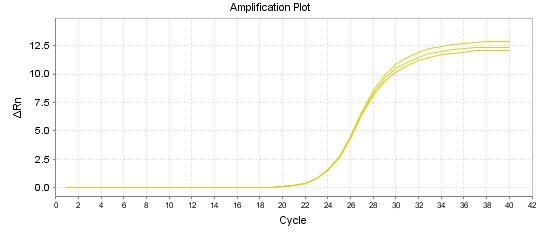

Supplement: S4 Data — (ZIP) [file pone.0140551.s004.zip › S4/COX2ú¿tnbs).jpg]

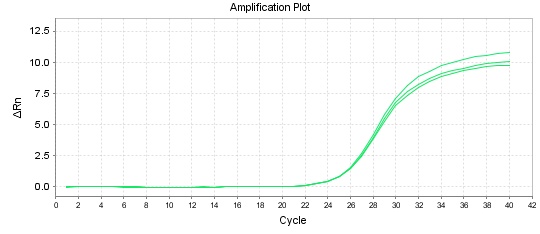

Supplement: S4 Data — (ZIP) [file pone.0140551.s004.zip › S4/INOS(100).jpg]

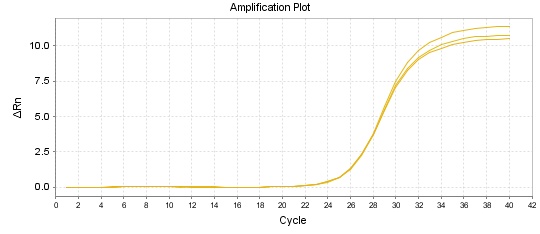

Supplement: S4 Data — (ZIP) [file pone.0140551.s004.zip › S4/INOS(200).jpg]

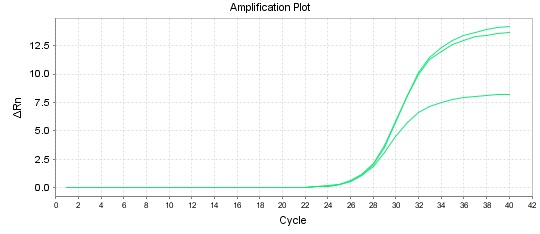

Supplement: S4 Data — (ZIP) [file pone.0140551.s004.zip › S4/INOS(50).jpg]

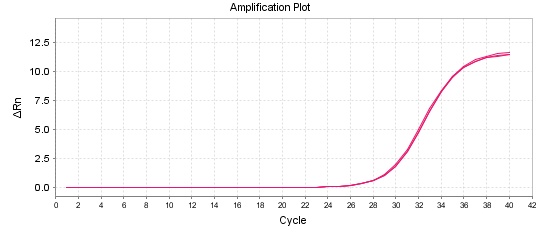

Supplement: S4 Data — (ZIP) [file pone.0140551.s004.zip › S4/INOS(control).jpg]

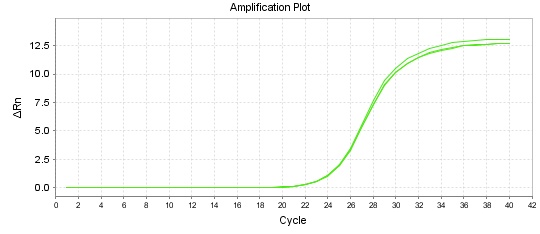

Supplement: S4 Data — (ZIP) [file pone.0140551.s004.zip › S4/INOS(tnbs).jpg]

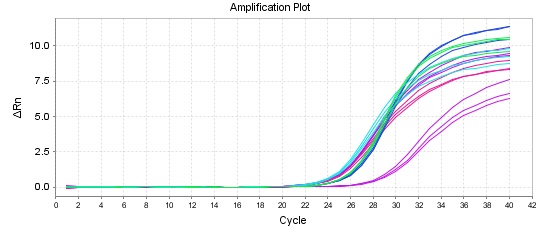

Supplement: S4 Data — (ZIP) [file pone.0140551.s004.zip › S4/INOS.jpg]

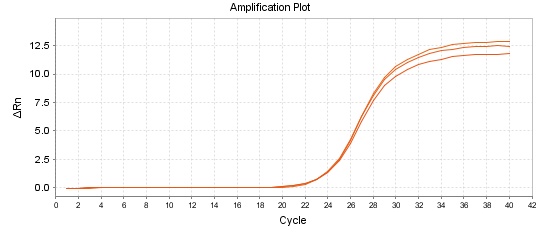

Supplement: S4 Data — (ZIP) [file pone.0140551.s004.zip › S4/P65(200).jpg]

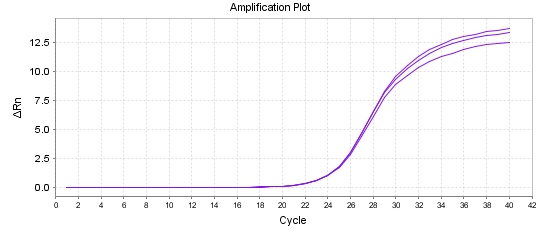

Supplement: S4 Data — (ZIP) [file pone.0140551.s004.zip › S4/P65(50).jpg]

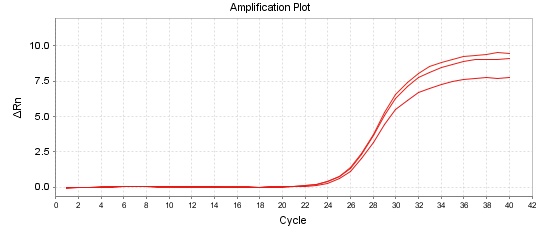

Supplement: S4 Data — (ZIP) [file pone.0140551.s004.zip › S4/P65(control).jpg]

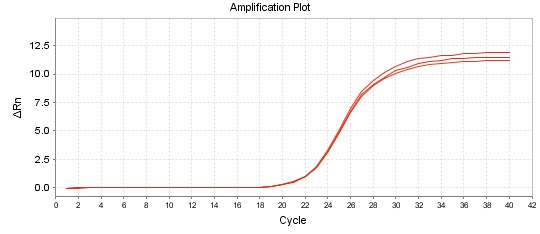

Supplement: S4 Data — (ZIP) [file pone.0140551.s004.zip › S4/P65(tnbs).jpg]

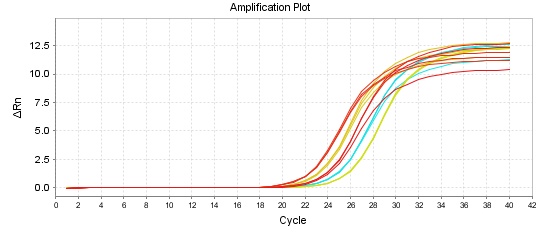

Supplement: S4 Data — (ZIP) [file pone.0140551.s004.zip › S4/P65.jpg]

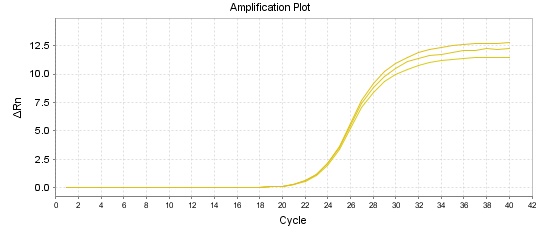

Supplement: S4 Data — (ZIP) [file pone.0140551.s004.zip › S4/P65ú¿100).jpg]

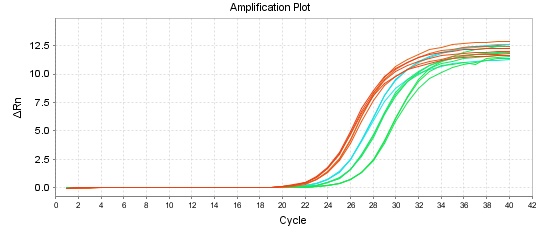

Supplement: S4 Data — (ZIP) [file pone.0140551.s004.zip › S4/TNFa.jpg]

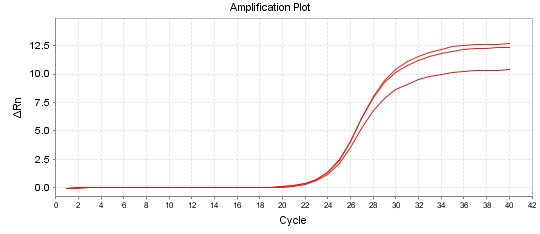

Supplement: S4 Data — (ZIP) [file pone.0140551.s004.zip › S4/TNFa┴(100).jpg]

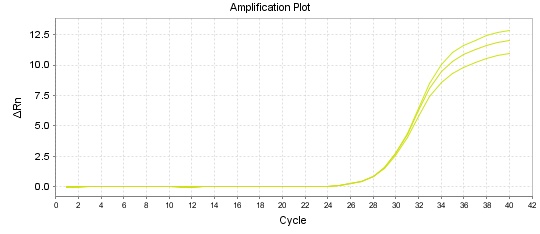

Supplement: S4 Data — (ZIP) [file pone.0140551.s004.zip › S4/TNFa┴(50).jpg]

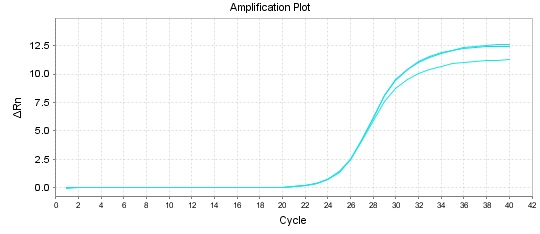

Supplement: S4 Data — (ZIP) [file pone.0140551.s004.zip › S4/TNFa┴ú¿200).jpg]

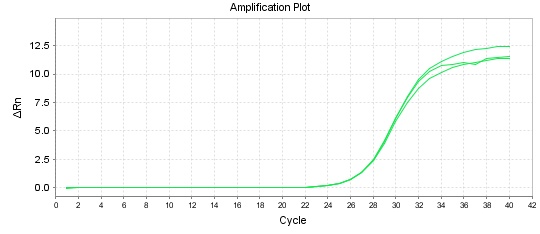

Supplement: S4 Data — (ZIP) [file pone.0140551.s004.zip › S4/TNFa┴ú¿control).jpg]

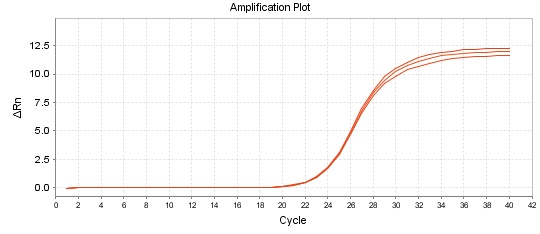

Supplement: S4 Data — (ZIP) [file pone.0140551.s004.zip › S4/TNFa┴ú¿tnbs).jpg]
